# Case for diagnosis. Disseminated erythematous and scaly plaques: chronic mucocutaneous candidiasis^☆^

**DOI:** 10.1016/j.abd.2022.08.013

**Published:** 2023-05-16

**Authors:** Nathalia Chebli de Abreu, Samuel Duarte Timponi França, Hyllo Baeta Marcelo Júnior, Amanda Neto Ladeira

**Affiliations:** a Department of Dermatology, Hospital Infantil João Paulo II, Fundação Hospitalar do Estado de Minas Gerais, Belo Horizonte, MG, Brazil; b Department of Dermatology, Hospital Eduardo de Menezes, Fundação Hospitalar do Estado de Minas Gerais, Belo Horizonte, MG, Brazil; c Department of Mycology, Fundação Ezequiel Dias, Belo Horizonte, MG, Brazil

Dear Editor,

A four-year-old male patient from a rural area had disseminated erythematous scaling plaques, some with thick adhered vegetative crusts since he was three years old (Fig. 1‒2). There was no deterioration in the general health status or relevant family history. The mother reported multiple previous hospitalizations due to pericarditis, pneumonia, and skin infections, in addition to episodes of oral and genital candidiasis.

**Figure 1:**
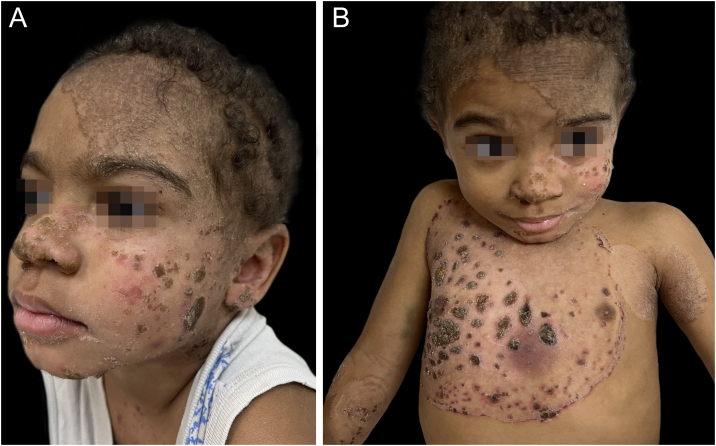
Erythematous scaling plaques with well-defined edges and thick adhered crusts on the face (A) and trunk (B)

**Figure 2:**
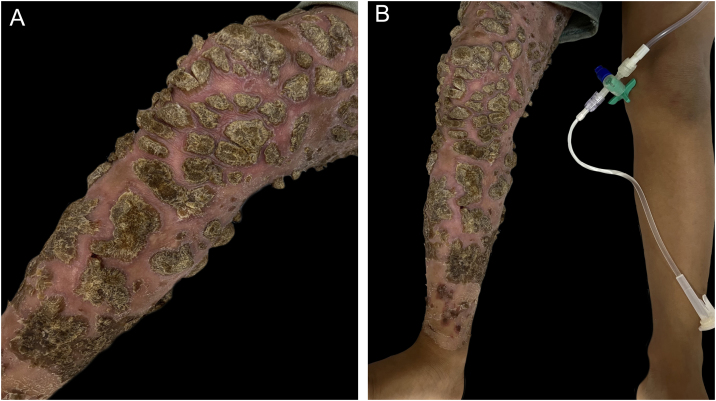
Thick vegetative crusts on erythematous plaques affecting the entire right lower limb (A‒B)

Direct mycological examinations of the skin lesions on the trunk and scalp disclosed the presence of hyphae, pseudohyphae, and yeasts – later identified as *Microsporum gypseum* and *Candida albicans* by MALDI-TOF (Matrix-assisted laser desorption ionization time-of-flight) mass spectrometry. Histopathology revealed irregular acanthosis, spongiosis, keratotic crust, and dermal edema, in addition to numerous hyphae and spores restricted to the stratum corneum (Fig. 3). The genome analysis identified a rare heterozygous mutation in exon 7 of the signal transducer and activator of transcription 1 (*STAT1*) gene; variant c.501A→C; p.Gln167His.

**Figure 3:**
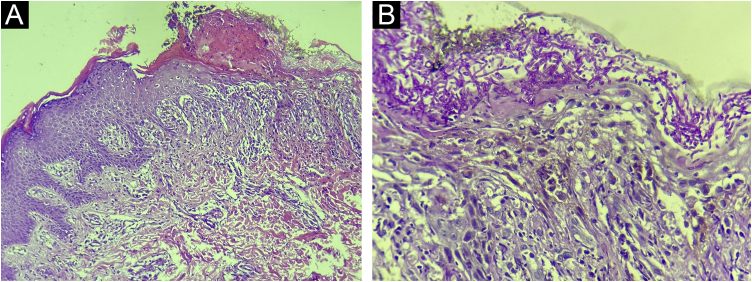
(A) Irregular acanthosis, spongiosis and keratotic crust are observed, in addition to dermal edema with areas of blurring of the dermal-epidermal junction (Hematoxylin & eosin, ×100). (B) Presence of numerous hyphae and spores in the stratum corneum (Periodic Acid-Schiff, ×400)

## What's your diagnosis?

a) Acquired Immunodeficiency Syndrome (AIDS)
b) Severe Combined Immunodeficiency (SCID)
c) Chronic Mucocutaneous Candidiasis (CMCC)
d) Hyper-IgE Syndrome (HIES)

## Discussion

**Figure 4:**
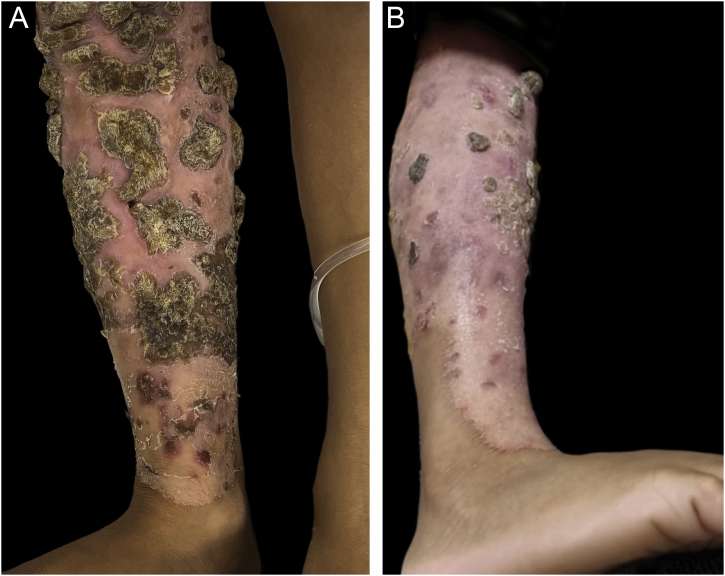
Partial regression of skin lesions. Aspect of the right lower limb before (A) and four weeks after starting fluconazole (B)

Based on the clinical-laboratory correlation, the diagnosis of chronic mucocutaneous candidiasis (CMCC) was established due to the *STAT1* gene mutation, in addition to extensive dermatophytosis. Complementary exams, including indirect Coombs, thyroid function, anti-HIV I and II serology, autoantibodies, immunoglobulin measurement and lymphocyte immunophenotyping were normal. Oral fluconazole was started with partial regression of the lesions (Fig. 4).

CMCC is a heterogeneous group of rare syndromes characterized by persistent, non-invasive *Candida spp* infections of the skin, nails, and mucous membranes caused by primary immunological defects.^1^ *STAT1* gain-of-function mutations underlie the autosomal dominant form of the disease and result in defective Th1 and Th17 cell responses, characterized by reduced production of interferon-γ, interleukin-17, and interleukin-22 cytokines, crucial for antifungal defense of the skin and mucous membranes.^2^, ^3^, ^4^ To the best of our knowledge, this is the first report in which the detected *STAT1* variant was documented in association with CMCC.

Typically, this form of the disease manifests as erythematous scaling crusted, hyperkeratotic generalized plaques before the age of five, sometimes accompanied by paronychia, hyperkeratosis and nail dystrophy. The oral mucosa is the most frequently affected, although the esophageal, genital and laryngeal mucosa can be affected as well. In addition to chronic *Candida* infection, there is also increased susceptibility to dermatophyte and bacterial infections, and up to 50% of the patients have associated hypothyroidism, inflammatory bowel disease, or associated autoimmune cytopenias.^5^, ^6^

The analysis of relevant genes, such as *STAT1*, *AIRE* and *CARD9*, is the only definitive laboratory test for the diagnosis of CMCC. Other immunodeficiencies, including SCID, HIES, and AIDS, can result in chronic candidiasis, but almost invariably course with invasive *Candida* infections and additional clinical-laboratory characteristics. In SCID, severe disturbances in T-, B-, and sometimes natural killer-cell development and function result in failure to thrive, chronic diarrhea, and recurrent severe infections with common viral pathogens (such as respiratory syncytial virus, adenovirus, and cytomegalovirus), and opportunistic microorganisms – which, in general, lead to death in the first year of life. HIES, in turn, is characterized by persistent generalized eczema, deep staphylococcal abscesses, *Aspergillus* infections, dimorphic features and recurrent fractures, in addition to increased levels of IgE, eosinophilia and mutation in the *STAT3* gene. Finally, AIDS is differentiated from CMCC by positive HIV serology, reduced CD4+ T-cell count, and occurrence of opportunistic infections.^6^, ^7^

Treatment of CMCC involves infection control and management of associated endocrine and autoimmune disorders. *Candida* infections can be controlled with prolonged use of azole antifungals, preferably fluconazole 100‒200 mg/day. Other therapies have been described in isolated reports to control the immune disorder, such as thymus and hematopoietic cell transplantation.^8^ Recently, some studies have reported good disease control with the use of JAK inhibitors, including ruxolitinib and baricitinib.^9^, ^10^

## Financial support

None declared.

## Authors' contributions

Nathalia Chebli de Abreu: Design and planning of the study; collection, analysis, and interpretation of data; critical review of the literature; drafting and editing of the manuscript.

Samuel Duarte Timponi France: Critical review of the literature; drafting and editing of the manuscript.

Hyllo Baeta Marcelo Júnior: Collection, analysis and interpretation of data; critical review of the literature; critical review of the manuscript.

Amanda Neto Ladeira: Design and planning of the study; collection, analysis, and interpretation of data; approval of the final version of the manuscript; intellectual participation in the propaedeutic and/or therapeutic conduct of the studied case.

## Conflicts of interest

None declared.

## References

[1] Kirkpatrick C.H. (1994). Chronic mucocutaneous candidiasis. J Am Acad Dermatol..

[2] Van de Veerdonk F.L., Plantinga T.S., Hoischen A., Smeekens S.P., Joosten L.A.B., Gilissen C. (2011). STAT1 mutations in autosomal dominant chronic mucocutaneous candidiasis. N Engl J Med..

[3] Eyerich K., Foerster S., Rombold S., Seidl H., Behrendt H., Hofmann H. (2008). Patients with chronic mucocutaneous candidiasis exhibit reduced production of Th17-associated cytokines IL-17 and IL-22. J Invest Dermatol..

[4] Van der Graaf C.A.A., Netea M.G., Drenth I.P.H., te Morsche R.H., van der Meer J.W.M., Kullberg B.J. (2003). Candida-specific interferon-gamma deficiency and toll-like receptor polymorphisms in patients with chronic mucocutaneous candidiasis. Neth J Med..

[5] Toubiana J., Okada S., Hiller J., Oleastro M., Gomez M.L., Becerra J.C.A. (2016). Heterozygous STAT1 gain-of-function mutations underlie an unexpectedly broad clinical phenotype. Blood..

[6] Kirkpatrick C.H. (2001). Chronic mucocutaneous candidiasis. Pediatr Infect Dis J..

[7] Egri N., Esteve-Solé A., Deyà-Martínez A., de Landazuri I.O., Vlagea A., García A.P. (2021). Primary immunodeficiency and chronic mucocutaneous candidiasis: pathophysiological, diagnostic, and therapeutic approaches. Allergol Immunopathol (Madr)..

[8] Van de Veerdonk F.L., Netea M.G. (2016). Treatment options for chronic mucocutaneous candidiasis. J Infect..

[9] Higgins E., Shehri T.A., McAleer M.A., Conlon N., Feighery C., Lilic D. (2015). Use of ruxolitinib to successfully treat chronic mucocutaneous candidiasis caused by gain-of-function signal transducer and activator of transcription 1 (STAT1) mutation. J Allergy Clin Immunol..

[10] Meesilpavikkai K., Dik W.A., Schrijver B., Nagtzaam N.M.A., Sluijs S.J.P., van Hagen P.M. (2018). Baricitinib treatment in a patient with a gain-of-function mutation in signal transducer and activator of transcription-1 (STAT1). J Allergy Clin Immunol..

